# Carboxymethyl Chitosan/Sodium Alginate/Chitosan Quaternary Ammonium Salt Composite Hydrogel Supported 3J for the Treatment of Oral Ulcer

**DOI:** 10.3390/gels9080659

**Published:** 2023-08-16

**Authors:** Tao Lin, Dandan Chen, Yan Geng, Jiayu Li, Yanghui Ou, Zhijun Zeng, Canqiang Yin, Xudong Qian, Xiang Qiu, Gang Li, Yali Zhang, Wen Guan, Mengjie Li, Xiaojia Cai, Jiaqiang Wu, Wen-Hua Chen, Yan-Qing Guan, Hongliang Yao

**Affiliations:** 1 School of Life Sciences, South China Normal University, Guangzhou 510631, China; 2022023039@m.scnu.edu.cn; 2 Guangdong Key Laboratory of Animal Conservation and Resource Utilization, Guangdong Public Laboratory of Wild Animal Conservation and Utilization, Institute of Zoology, Guangdong Academy of Sciences, Guangzhou 510260, China; chen15188581741@163.com (D.C.); m18797822920@163.com (Y.G.); m18344049665@163.com (J.L.); ouyh0807@gmail.com (Y.O.); jessica_zengzj@163.com (Z.Z.); yincanqiang123@163.com (C.Y.); qianxudong0228@163.com (X.Q.); qxiang5202023@163.com (X.Q.); ligang00890089@163.com (G.L.); zhangyl@giz.gd.cn (Y.Z.); guanwen310@126.com (W.G.); lmj100800@163.com (M.L.); 3 School of Biotechnology and Health Sciences, Wuyi University, Jiangmen 529020, China; chemicalcxj@163.com (X.C.); wyuchemwjq@wyu.edu.cn (J.W.); whchen@wyu.edu.cn (W.-H.C.)

**Keywords:** oral ulcer, hydrogel, carboxymethyl chitosan, chitosan quaternary ammonium salt, sodium alginate

## Abstract

Oral ulcer is a common inflammatory disease of oral mucosa, causing severe burning pain and great inconvenience to daily life. In this study, compound **3J** with anti-inflammatory activity was synthesized beforehand. Following that, an intelligent composite hydrogel supported **3J** was designed with sodium alginate, carboxymethyl chitosan, and chitosan quaternary ammonium salt as the skeleton, and its therapeutic effect on the rat oral ulcer model was investigated. The results show that the composite hydrogel has a dense honeycomb structure, which is conducive to drug loading and wound ventilation, and has biodegradability. It has certain antibacterial effects and good anti-inflammatory activity. When loaded with **3J**, it reduced levels of TNF-α and IL-6 in inflammatory cells by up to 50.0%. It has excellent swelling and water retention properties, with a swelling rate of up to 765.0% in a pH 8.5 environment. The existence of a large number of quaternary ammonium groups, carboxyl groups, and hydroxyl groups makes it show obvious differences in swelling in different pH environments, which proves that it has double pH sensitivity. It is beneficial to adapt to the highly dynamic changes of the oral environment. Compared with single hydrogel or drug treatment, the drug-loaded hydrogel has a better effect on the treatment of oral ulcers.

## 1. Introduction

An oral ulcer, also known as recurrent aphthous stomatitis (RAS), is a common chronic inflammatory injury disease that occurs in the oral mucosa. It is manifested by inflammation and oral mucosal injury. In the early stages of the disease, the affected area will show red spots accompanied by a burning or tingling sensation. As the disease progresses, ulcers begin to appear and cover the wound with a thick yellowish-white membrane of pus [1,2,3]. The etiology of oral ulcers may involve many factors such as genetics, physical injury, systemic disease, stress, vitamin and trace element deficiency, and infection [4]. In summary, apart from the two major factors of unchangeable heredity and careless bite, the remaining major influencing factors can be classified into three aspects: nutrition, immunity, and oral flora [5,6,7]. According to the different causes, it is clinically divided into recurrent oral ulcers, traumatic ulcers, cancerous ulcers, and infection-induced ulcers which are the most common four categories. At present, the most common treatment methods for oral ulcers include oral drugs, external gel or film application, and spray powder. However, different treatment methods have some shortcomings, for example, oral drugs have a first-pass effect and reduce drug bioavailability. Spraying powder is prone to ingestion [8,9]. The externally applied gel or film material contains a large amount of water and has its own flexibility and adaptability to human soft tissues. Moreover, it can also block the erosion of oral saliva and oral flora on the ulcer wound, alleviate pain, repair and promote the healing of ulcer wound, and isolate the affected area, thus becoming one of the most commonly used and efficient treatments for oral ulcers [10]. In addition, the moist and highly dynamic oral environment also makes the local treatment of oral ulcers challenging. Therefore, a hydrogel with viscosity, bacteriostasis, and adaption to oral environment changes is needed to assist the treatment.

Carboxymethyl chitosan (CMCS) is an important water-soluble chitosan derivative. It has strong antibacterial properties, promoting wound healing, moisture absorption and water retention, water solubility, biodegradability, and biocompatibility. Therefore, it is widely used in the development of multi-functional wound dressings. The carboxymethylation of chitosan improves water solubility, making it an effective carrier for the delivery of different drugs, especially those with poor water solubility, thus improving drug bioavailability [11,12]. Qiao et al. [13], based on oxidized sodium alginate grafted dopamine/carboxymethyl chitosan/Fe^3+^, prepared a self-healing hydrogel wound dressing. The hydrogel showed excellent antibacterial, hemostatic, and antioxidant capabilities. For oral surgery, a nanofiber membrane that is crosslinked with carboxymethyl chitosan and carrageenan can be utilized to achieve quick hemostasis [14]. A dressing made of carboxymethyl chitosan and starch, cross-linked with glyoxal, has been found to be effective in preventing the bleeding of oral tissues resulting from oral surgery [15].

Chitosan quaternary ammonium salt (AmCS) is a kind of chitosan derivative prepared by chemical modification. It is biocompatible, biodegradable, water-soluble, and highly hygmoscopic. It has strong tissue adhesion ability, bacteriostasis, anti-inflammatory activity, film-formation and wound healing functions, and is also one of the commonly used components of wound dressing [16,17,18]. A pH-sensitive hydrogel that promotes wound healing can be created by crosslinking the amino group in chitosan quaternary ammonium salt and the aldehyde group in oxidized dextran dopamine [19]. Zhang and his colleagues successfully prepared a gel film loaded with gentamicin sulfate by reacting chitosan quaternary ammonium salt with epichlorohydrin. The hydrogel has the potential value of excellent antibacterial wound dressing [20].

Based on the above properties of carboxymethyl chitosan and chitosan quaternary ammonium salt, they meet the basic requirements of moisturizing and antibacterial properties of gel dressings and have the advantages of anti-inflammatory and promoting wound healing. Therefore, they are selected as the framework for preparing hydrogels. In addition, the hydrogel dressing should also have a certain mechanical strength, that is, there is a cross-linked structure. In the presence of cations such as Ca^2+^ and Sr^2+^ [21], the Na^+^ on the G unit of sodium alginate (SA) can undergo ion exchange reaction with bivalent cations. And then the G unit can accumulate to form a cross-linked network structure, which enables SA to quickly form a gel under extremely mild conditions. Therefore, SA is often used to enhance the toughness and strength of hydrogels [22,23]. At the same time, SA contains a large amount of -COO^−^. Therefore, it can show polyanion behavior when dissolved in water, that is, it has a certain adhesion which can increase the viscosity of the gel. This also makes sodium alginate obtain obvious pH-sensibility [24,25,26,27,28].

Because CHF_2_ moiety can affect the acidity, lipophilicity, and metabolic stability of bioactive compounds, the difluoromethyl-containing compounds have a wide range of applications in medicine, agricultural chemicals, and other fields. Compounds that contain difluoromethyl, particularly those with α,α-difluoromethyl carbinols and structural motifs, have garnered significant interest for their potential as bioactive compounds and pharmaceuticals. Currently, common α,α-difluoromethyl carbinols drugs include gemcitabine [29], GABAB receptor agonist [30], JAK3 inhibitors [31], etc. These drugs all have good anti-inflammatory activity. **3J** (relative molecular weight of 271.2, insoluble in water) also belongs to α,α-difluoromethyl carbinols compounds, therefore it is speculated that they also have anti-inflammatory activity [32].

Therefore, in this study, a multi-level composite hydrogel was prepared using carboxymethyl chitosan, chitosan quaternary ammonium salt and sodium alginate as the matrix, and CaCl_2_ as the crosslinking agent. And the oral ulcer rat model was induced by phenol to explore the therapeutic effect of the hydrogel. Chitosan quaternary ammonium salts contain a large number of quaternary ammonium groups, while carboxymethyl chitosan and sodium alginate contain abundant carboxyl groups. When the hydrogel is in a highly acidic environment, the carboxyl group fails to ionize, causing the polymer chain to shrink. When the pH value of the environment surrounding the hydrogel is between subacidity and alkalescence, the swelling rate of the hydrogel increases significantly with an increase in pH value. The action of amino groups is opposite to that of carboxyl groups. The hydrogel materials’ dual pH-sensitivity and pharmacological activity are the main highlights of the study.

## 2. Results and Discussion

### 2.1. Preparation of Compound 3J

**Figure 1:**
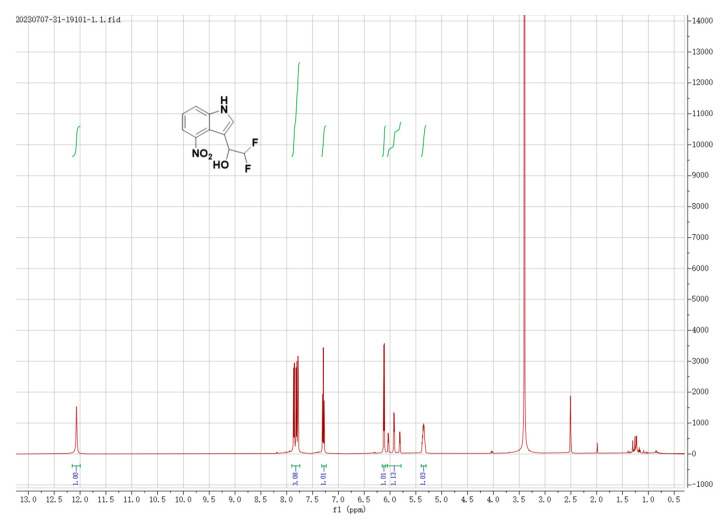
The ^1^H NMR results of **3J**. The green vertical line indicates that the peak is integrated, and the blue number is the size of the peak area accumulated by the corresponding peak.

Compound **3J**, that is, 2,2-difluoro-1-(4-nitro-1h-indole-3-yl)ethyl-1-ol), was prepared in 76.0% from the reaction of Friedel-Crafts with electron-rich arenes and 2,2-difluorovinyl arylsulfonates in trifluoroethanol, according to reported protocols [32]. Its structure was confirmed by means of ^1^H NMR data (Figure 1).

^1^H NMR (500 MHz, DMSO-*d*_6_) δ 12.07 (s, 1H), 7.95–7.72 (m, 3H), 7.29 (t, *J* = 7.9 Hz, 1H), 6.12 (d, *J* = 5.8 Hz, 1H), 5.92 (td, *J* = 56.2, 4.3 Hz, 1H), and 5.36 (t, *J* = 10.1, 5.1 Hz, 1H).

### 2.2. Hydrogel Preparation, Characterization, and Drug Release

#### 2.2.1. Hydrogel Preparation and Characterization

**Figure 2:**
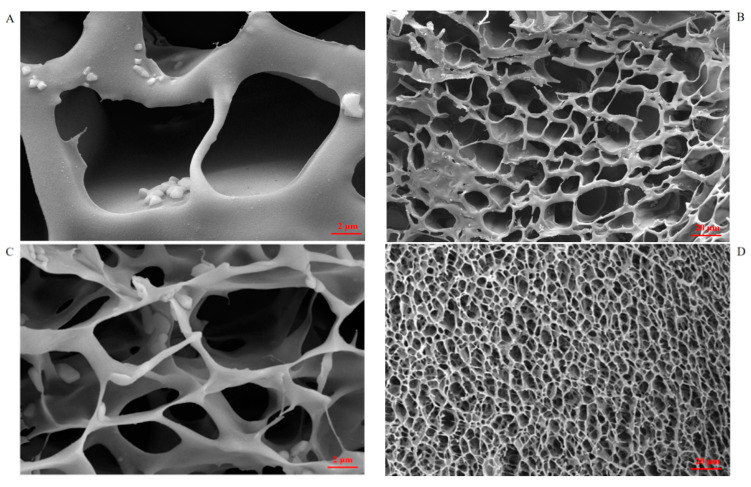

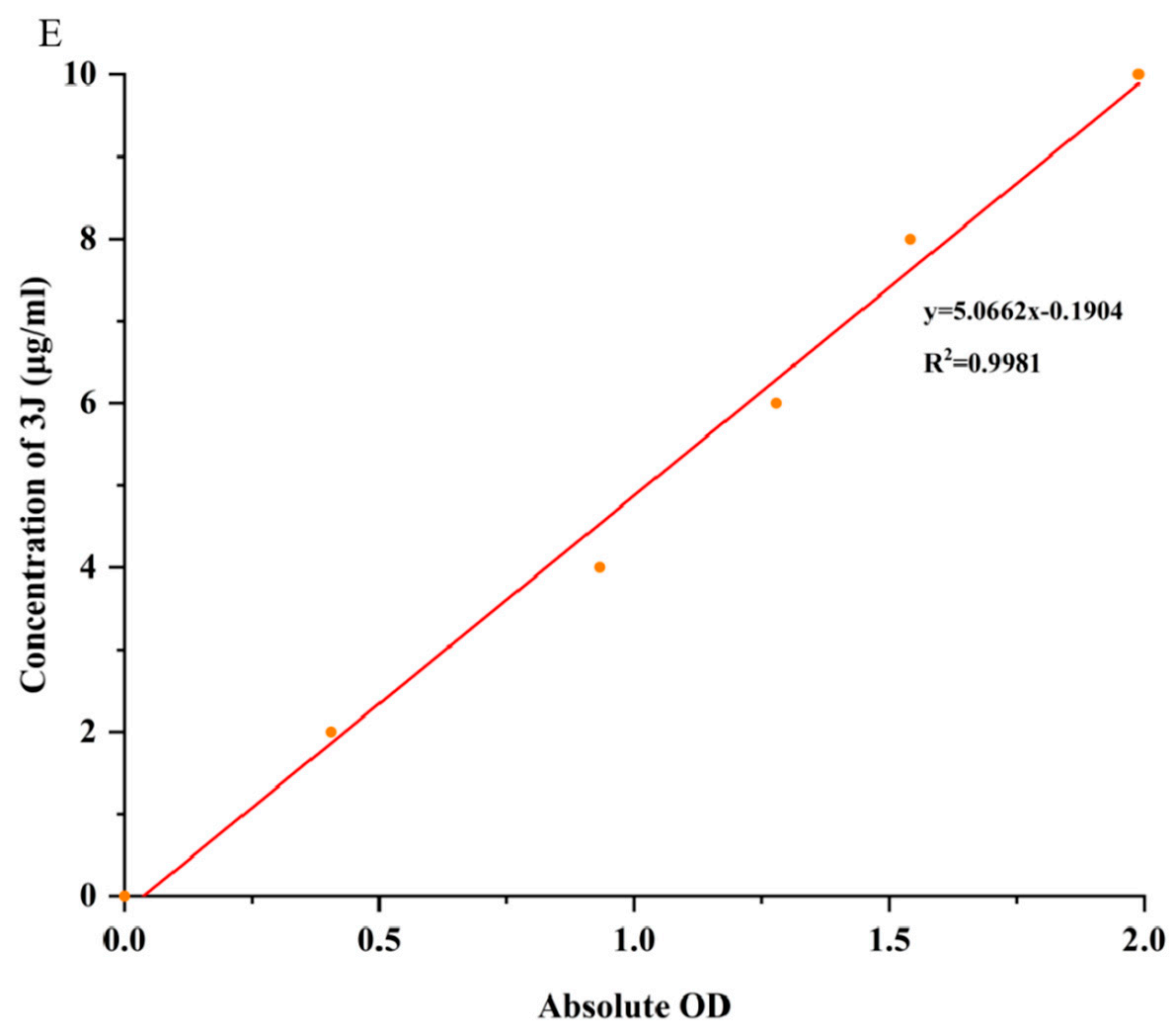
SEM morphological characteristics of composite hydrogels. (**A**) AmCS−SA hydrogel form under 2.0 microns. (**B**) AmCS−SA hydrogel form under 20.0 microns. (**C**) CMCS−SA−AmCS−SA hydrogel form under 2.0 microns. (**D**) CMCS−SA−AmCS−SA hydrogel form under 20.0 microns. (**E**) Standard curve of **3J**.

As can be seen from the AmCS-SA profile (Figure 2), the hydrogel composed only of chitosan quaternary ammonium salt and SA crosslinked by CaCl_2_ has large pores and disordered void arrangement, which may result in greater fluidity of the whole hydrogel. Moreover, the composite hydrogel CMCS-SA-AmCS-SA formed after adding CMCS, and the SA content improved the cross-linking degree, reduced the cavity size, and made the overall compact network structure similar to a cake. These advantages are beneficial for increasing drug loading and achieving drug sustained release [33], as well as promoting wound healing. Through UV full wavelength scanning, the maximum absorption wavelength of **3J** was determined at 245.0 nm, and a standard curve (Figure 2E) was drawn to convert the drug content in the sample. It was found that the drug loading rate and entrapment efficiency of multiple crosslinked hydrogel CMCS-SA-AmCS-SA can reach 9.56% ± 0.04% and 95.64% ± 0.01% (mean ± SD, *n* = 3).

**Figure 3:**
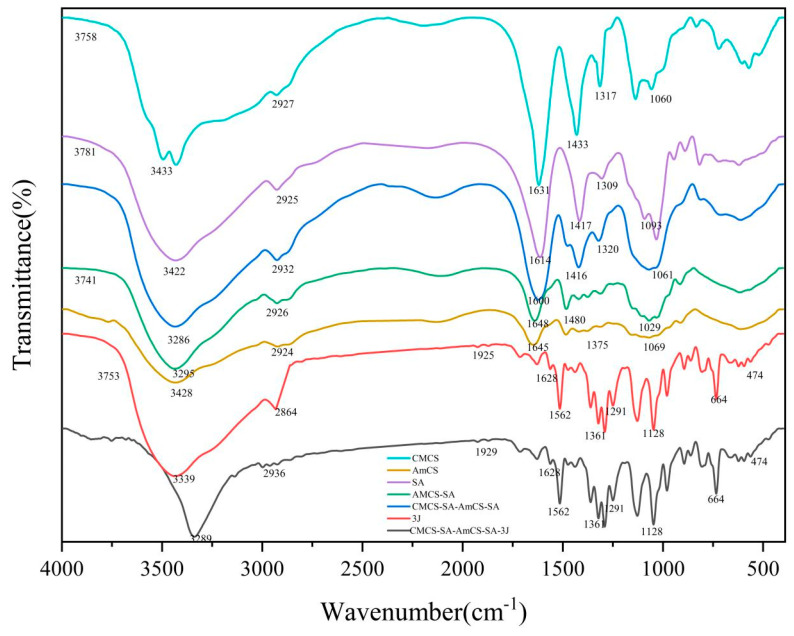
FT-IR spectra of CMCS, AmCS, SA, **3J**, AmCS−SA, CMCS−SA−AmCS−SA, and CMCS−SA−AmCS−SA−**3J**.

The FT-IR results in Figure 3 show that OH and NH_2_ tensile peaks appear near 3500.0–3000.0 cm^−1^ for both CMCS and AmCS, C-H tensile peaks appear at 2927.0 cm^−1^, and C-O-C tensile peaks appear at 1250.0 cm^−1^. SA shows an absorption band around 3500.0–3000.0 cm^−1^ due to its rich hydroxyl groups. The secondary cross-linking of sodium alginate and CMCS in CMCS-SA-AmCS-SA may cause partial or complete removal of acetyl group of CMCS, resulting in the weakening or the disappearance of acetyl group peak. The peak near 1600.0–1750.0 cm^−1^ indicates that deacetylation may have occurred [34]. The sharp peak of CMCS-SA-AmCS-SA hydrogel at 3286.0 cm^−1^ indicates that the SA content increases, more hydroxyl groups are introduced, the hydroxyl peak is significantly enhanced, and the hydrophilicity is increased [35]. The infrared spectrum of CMCS-SA-AmCS-SA hydrogel shows that it is rich in carboxyl (1600.0 cm^−1^), amino (3286.0 cm^−1^), and hydroxyl (3741.0 cm^−1^). This result preliminarily proves that the hydrogel is pH sensitive. The appearance of the characteristic peak of the difluoromethyl group at 13,500.0–1100.0 cm^−1^ confirms the successful synthesis of **3J**. After comparing CMCS-SA-AmCS-SA with CMCS-SA-AmCS-SA-**3J**, no new characteristic peaks were found in the latter. This confirms that **3J** is only physically loaded into CMCS-SA-AmCS-SA.

**Figure 4:**
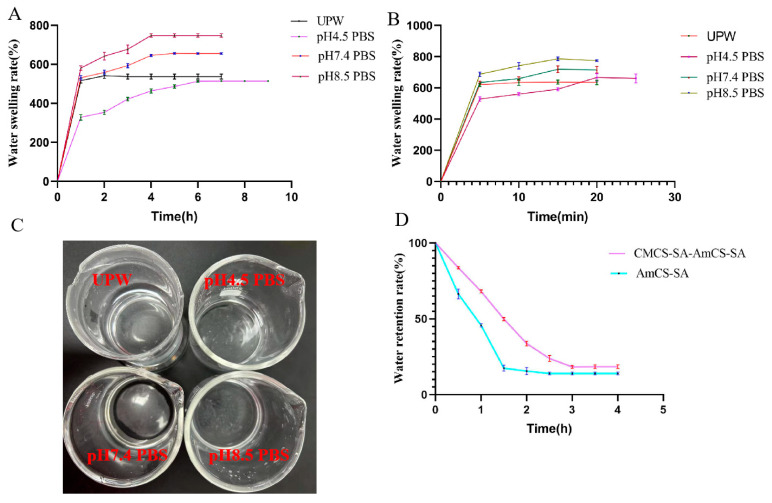
Characterization of swelling and water retention of hydrogels. (**A**) Swelling properties of CMCS−SA−AmCS−SA hydrogel (mean ± SD, *n* = 3). (**B**) Swelling properties of AmCS−SA hydrogel (mean ± SD, *n* = 3). (**C**) AmCS−SA dissolution results within 1 h. (**D**) Water retention of CMCS−SA−AmCS−SA and AmCS−SA (mean ± SD, *n* = 3).

Based on the findings in Figure 4A, the CMCS-SA-AmCS-SA composite hydrogel displays the quickest water absorption within 0.5 h across various pH conditions. After that, its water absorption rate is still increasing (the growth rate is small) and gradually tends to be stable until it reaches the equilibrium state of swelling. And pH-responsive hydrogel is a type of smart hydrogel that experiences a significant change in swelling rate according to pH levels. Typically, the hydrogel’s structure contains polar groups, such as amino, carboxyl, and hydroxyl groups. The CMCS-SA-AmCS-SA hydrogel exhibits noticeable differences in swelling depending on the pH level, which can be attributed to its abundance of polar groups such as carboxyl, amino, and hydroxyl groups. This finding is consistent with the results gathered from infrared spectroscopy, which confirms its pH-responsive properties. The hydrogel responds to changes in environmental pH via protonation and deprotonation of the carboxyl and amino groups, leading to a phase transition. In addition, due to the higher proportion of carboxyl in CMCS-SA-AmCS-SA, its swelling rate is higher in a slightly alkaline environment, which is more conducive to the role of hydrogel in the ulcer site of rats with oral ulcers (the pH of this site measured in the experiment is 7.4–8.5).

AmCS-SA absorbed water within 5 min after drying and showed differences in swelling at different pH levels (Figure 4B). In particular, it can be seen in Figure 4B,C that AmCS-SA degrades partially within 20 min and completely within an hour. This result may be due to the fact that, as reported in the literature, chitosan quaternary ammonium salt has very good solubility [13]. Moreover, the cross-linking effect of SA and CaCl_2_ is weak. As shown in Figure 2A,B, the internal structure of AmCS-SA is loose, and during the drying process at 50 °C, the cross-linking structure between each component is further destroyed, resulting in poor swelling and easy dissolution. With the addition of carboxymethyl chitosan and the increase of SA content, the cross-linking degree of CMCS-SA-AmCS-SA composite hydrogel is stronger than AmCS-SA, so the swelling property is improved, and the water retention of CMCS-SA-AmCS-SA is stronger than AmCS-SA (Figure 4C).

**Figure 5:**
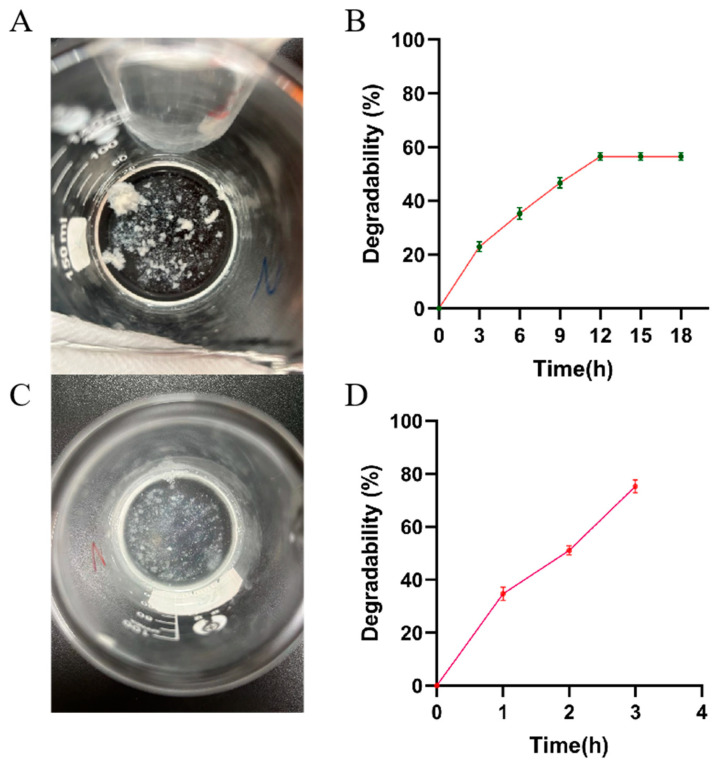
Degradation rate characterization of (**A**,**B**) CMCS−SA−AmCS−SA hydrogel and (**C**,**D**) AmCS−SA hydrogel.

The two hydrogels, CMCS-SA-AmCS-SA and AmCS-SA, were immersed in PBS buffer with pH7.4 and placed in an incubator at 37 °C. Both CMCS-SA-AmCS-SA and AmCS-SA began to degrade rapidly when they reached swelling equilibrium. Figure 5A,C show the states of CMCS-SA-AmCS-SA and AmCS-SA, respectively, after only 20 min. It is evident that the degradation rate of CMCS-SA-AmCS-SA is lower than that of AmCS-SA. Additionally, the degradation rate remains almost constant after reaching about 60% (Figure 5B). This result, together with the swelling result, proves that the improvement of crosslinking degree in hydrogel can enhance the strength of hydrogel and reduce the degradation rate. This feature is useful for achieving a sustained drug release and adapting to the dynamic and complex environment of the oral cavity. Specifically, when AmCS-SA that dried at 50 °C was immersed in PBS buffer at pH 7.4, it completely degraded within 1 h (Figure 4C). However, the freeze-dried AmCS-SA was almost completely degraded after soaking for 3 h (Figure 5C,D). This indicates that the method of drying will impact the physical and chemical properties of the hydrogel.

**Figure 6:**
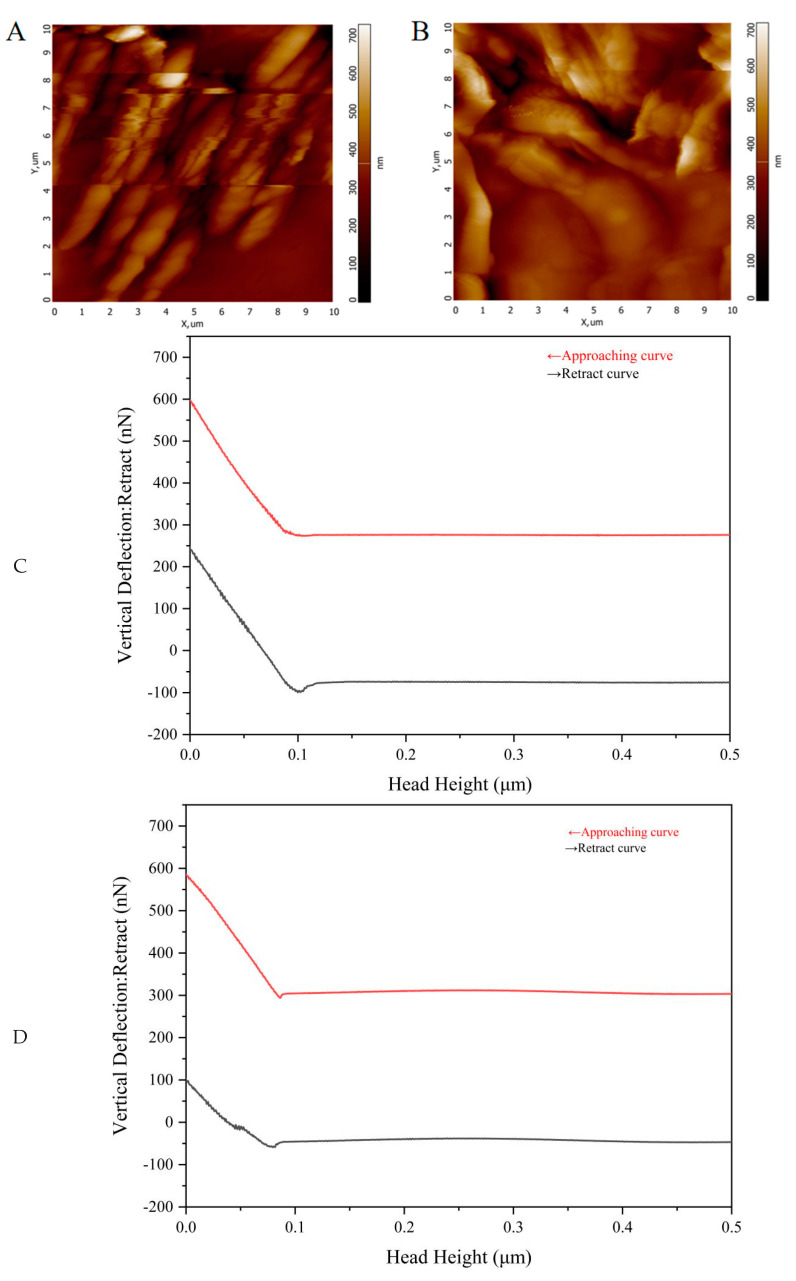
Rheological characterization of hydrogels. (**A**) Surface morphology imaging of AmCS−SA hydrogel under atomic force microscope (AFM). (**B**) Surface morphology imaging of CMCS−SA−AmCS−SA hydrogel under AFM. (**C**,**D**) The determination process of Young’s modulus of (**C**) AmCS−SA hydrogel and (**D**) CMCS−SA−AmCS−SA hydrogel.

Figure 6A,C show the topography and Young’s modulus measurement of AmCS-SA. It can be seen that when the applied Young’s modulus elasticity is 2238.1 MPa, its minimum and maximum deformations are 27.92 nm and 271.45 nm, respectively. From the length of the deformation, we can see that its pressure resistance and rigidity are relatively strong. Hydrogel has a larger elastic modulus, so its resistance to external stress is stronger. According to the measurement diagram of CMCS-SA-AmCS-SA in Figure 6D, when the applied pressure is 707.7 MPa, the corresponding minimum and maximum deformations are 63.67 nm and 328.94 nm, indicating that this hydrogel has moderate rigidity and can maintain structural stability. The release of the drug can be controlled within the required range of time. The morphology images under AFM do not reflect the exact surface morphology but can show the geographical shape of the hydrogel surface. We scanned and imaged AmCS-SA and CMCS-SA-AmCS-SA at a resolution of 256 × 256 pixels. From Figure 6A, it can be seen that the maximum peak height on the surface of AmCS-SA is 700.0 nm. At the same height, CMCS-SA-AmCS-SA presents a full view, indicating that its highest peak is below 700.0 nm. These results are consistent with the SEM results, indicating that the internal pores of AmCS-SA are large and the section is irregular, while the internal pores of CMCS-SA-AmCS-SA are small and the structure is dense.

#### 2.2.2. Drug Release from Hydrogels

**Figure 7:**
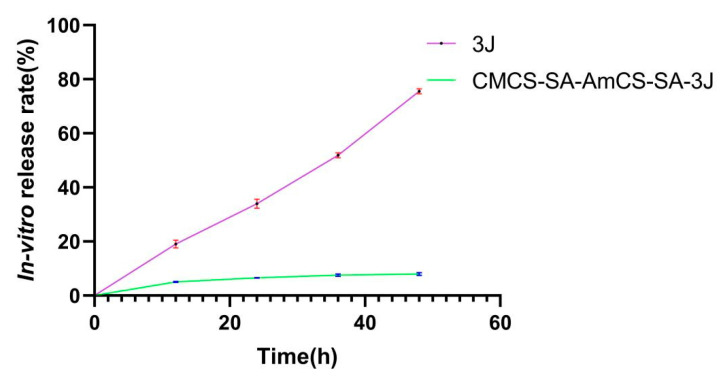
In vitro release of free **3J** and **3J** loaded CMCS−SA−AmCS−SA in a PBS buffer solution (pH 7.4) at 37 °C for 48 h (mean ± SD, *n* = 3).

It is necessary to maintain a relatively constant drug concentration at the lesion site to accelerate the healing of the ulcer site. It is obvious from the two drug release curves in Figure 7 that the drug release rate is significantly slowed down after hydrogel encapsulation, which indicates that the composite hydrogel can play a role in helping slow release. Based on the data obtained from the infrared spectrum, it has been determined that there is no chemical interaction between **3J** and CMCS-SA-AmCS-SA. Instead, **3J** is only physically loaded into the cavity of the CMCS-SA-AmCS-SA composite hydrogel. In addition, due to the extremely high degree of crosslinking of CMCS-SA-AmCS-SA, the internal structure is dense and the pores are small, resulting in the slow release of **3J**, which is consistent with the results in Figure 7.

### 2.3. In Vivo Antibacterial Properties

**Figure 8:**
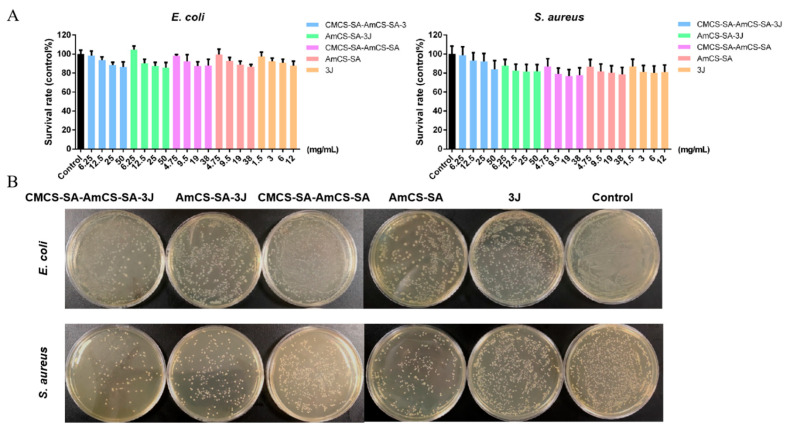
Evaluation of the antibacterial effect of hydrogels. (**A**) Survival ratio was calculated via OD_600_ for *S. aureus* and *E. coli* (mean ± SD, *n* = 3). (**B**) Images of survival bacteria clones after the treatment of hydrogels.

The oral environment is complex, in which many microorganisms live, and oral flora is closely related to the recovery of oral ulcers. The infection of *Staphylococcus aureus*, candida, and other microorganisms, as well as the imbalance of oral flora, can lead to the occurrence of oral ulcers or prolong the recovery time of ulcers [36,37,38]. Therefore, the preparation of hydrogels dressings with antibacterial properties is very necessary. And the antibacterial properties of the hydrogels were assessed via optical density (OD) at 600.0 nm (OD_600_). As shown in Figure 8A, we first explored the antibacterial effects of different concentrations of AmCS-SA and the composite CMCS-SA-AmCS-SA hydrogels. The results showed that both of them had a certain antibacterial effect on the growth of *Escherichia coli* (ATCC 25922) and *Staphylococcus aureus* (ATCC 25923). The antibacterial outcome was enhanced gradually as the concentration increased, and the antibacterial impression of CMCS-SA-AmCS-SA was slightly stronger than that of AmCS-SA at the same concentration. In other words, carboxymethyl chitosan was added to AmCS-SA hydrogel and the concentration of sodium alginate was increased, and the antibacterial effect of AmCS-SA hydrogel was enhanced by further coating. According to previous reports, the reason for this result may be that the introduction of -COOH in carboxymethyl chitosan causes more -NH_2_ to become -NH_3_^+^, thus increasing the number of disinfection factors. Both chitosan quaternary ammonium salt and sodium alginate can bind with protein and sugar molecules on the surface of bacteria to form a protective layer, preventing further growth and reproduction of bacteria. A small number of amide bonds formed during the preparation of hydrogels may also be a factor promoting antibacterial activity [10,13,39]. As can be seen from the figure, small molecule drug **3J** and two kinds of hydrogels loaded with drugs also have similar antibacterial results, but their combination did not significantly enhance the antibacterial effect. This result may be attributed to the dense pore structure inside the hydrogel, which slows down the drug release rate, so the antibacterial effect of the hydrogel loaded with drugs does not improve significantly in a short time. At low concentrations, each hydrogel had almost no inhibitory effect on *Escherichia coli*, and even AmCS-SA and AmCS-SA-**3J** promoted its growth. It may be related to the excellent water solubility of chitosan quaternary ammonium salts. At very low concentrations, a small number of polysaccharides decomposed into monosaccharides to provide carbon sources for the growth of *Escherichia coli*. The results of lipid plate counting in Figure 8B are comparable to OD_600_, further verifying the results in Figure 8A.

### 2.4. Anti-Inflammatory Effects of Hydrogels and 3J

**Figure 9:**
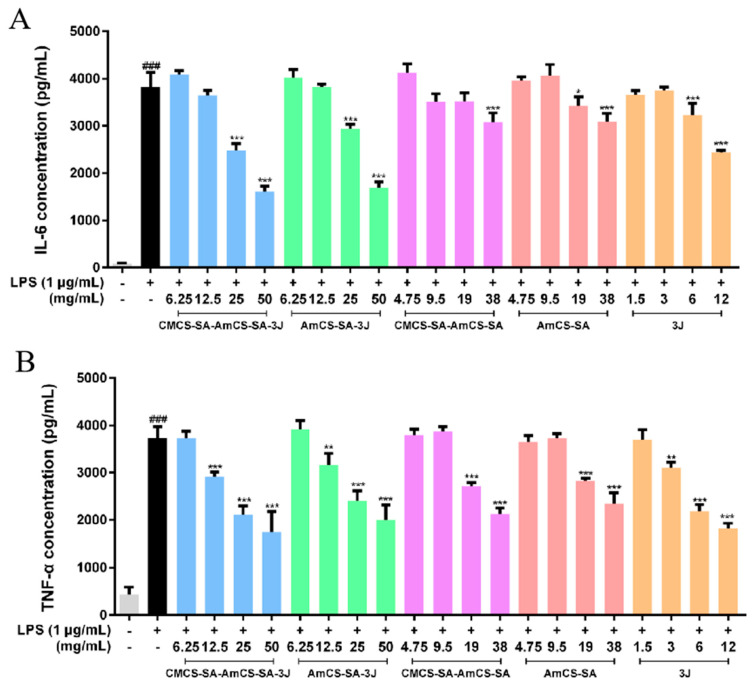
Anti-inflammatory properties of hydrogels and **3J** in vitro. (**A**) IL-6 and (**B**) TNF-α were detected by an enzyme-linked immunosorbent assay (ELISA) (mean ± SD, *n* = 3) vs. LPS, * *p* < 0.05, ** *p* < 0.01, *** *p* < 0.001; vs. control, ### *p* < 0.001.

During the occurrence and recovery of oral ulcers, a large number of cytokines and chemokines, such as TNF-α, IL-1β, IL-6, and IL-8, were involved in the regulation of inflammatory response and affect the process of oral ulcers. Among these inflammatory factors, TNF-α and IL-6 play a leading role [40,41,42,43]. Therefore, macrophages (RAW 264.7) were incubated in diluents of **3J** or different hydrogels after inflammation induction with Lipopolysaccharide, and the levels of TNF-α and IL-6 were used as evaluation indexes to investigate their anti-inflammatory effects. As shown in Figure 9, ELISA results show that both **3J** and various hydrogel samples can reduce the strengths of TNF-α and IL-6. The result indicates that **3J** and various hydrogel samples can mediate the weakening of macrophage inflammatory response. And in a certain range, with the increase of drug concentration, their anti-inflammatory effect was significantly enhanced. Our results further indicate that the anti-inflammatory effect of hydrogels loaded with drugs is better than that of drugs or hydrogels alone, and that the anti-inflammatory effect of CMCS-SA-AmCS-SA-**3J** is the best.

### 2.5. In Vivo Efficacy Assay

**Figure 10:**
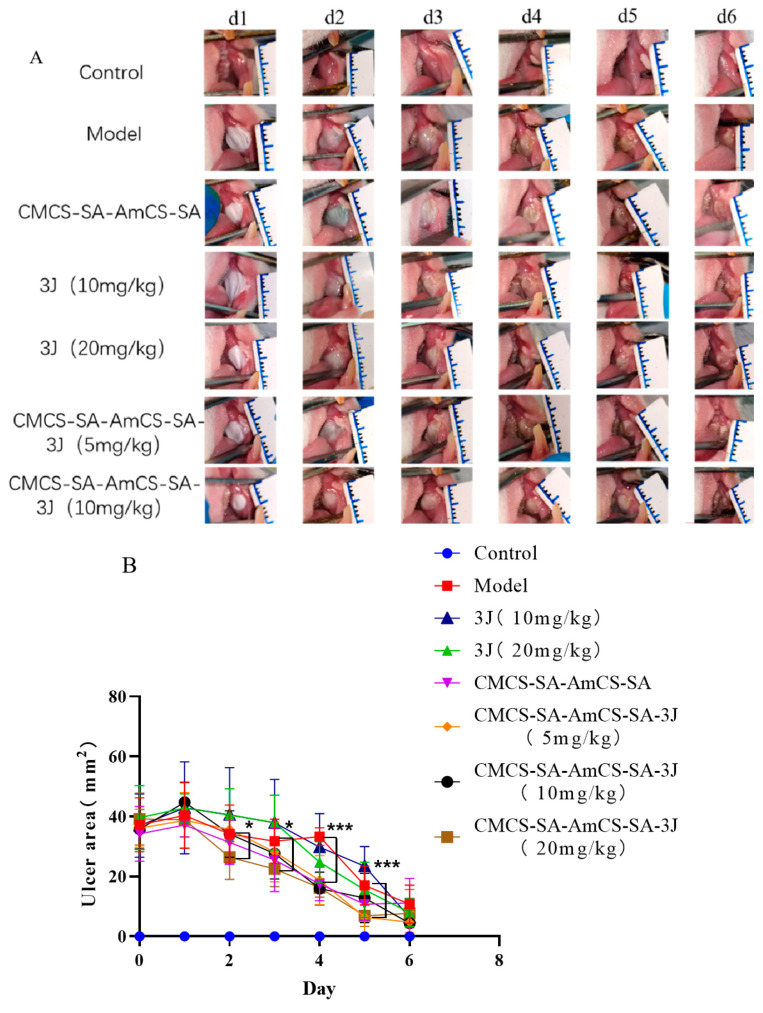

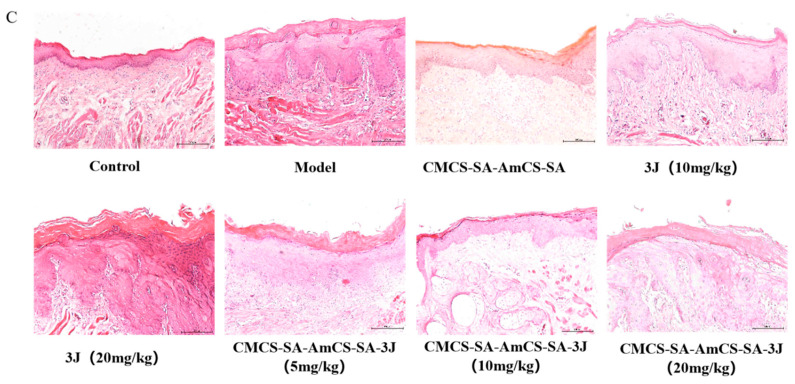
Analysis of the effect of treating oral ulcer in vivo. (**A**) Changes of ulcer area in each group at 0−6 days after establishing oral ulcer model induced by phenol (mean ± SD, *n* = 8). (**B**) Quantitative results of oral ulcer area at different time points (mean ± SD, *n* = 8), * *p* < 0.05, *** *p* < 0.001. (**C**) HE staining of the oral mucosa of SD rats (magnification 100×).

To investigate the effect of CMCS-SA-AmCS-SA and **3J** on oral ulcer healing in vivo, a rat model of oral ulcer was established by phenol induction and treated by CMCS-SA-AmCS-SA or **3J** for 5 days. Therefore, the ulcer area in each group was measured every day. As can be seen in Figure 10A, the oral mucosa of rats turned white on the second day after modeling, forming a certain pseudomembrane, with red and swollen edges and signs of suppuration on the surface. As illustrated in Figure 10B, with the extension of administration time, the area of ulcer was decreased in every group. Compared to the other groups, CMCS-SA-AmCS-SA hydrogel and CMCS-SA-AmCS-SA-**3J** (20 kg/mg) hydrogel showed the best therapeutic effect.

Histological evaluations of the oral ulcer samples on day 6 are presented in Figure 10D. Oral mucosal epithelial cells of rats in control group were intact without bleeding and ulcer lesions. Oral mucosal epithelium of rats in model group was necrotic and exfoliated, and a large number of inflammatory cells infiltrated the laminae propria, resulting in inflammation. Compared with the model group, oral mucosal lesions in the other groups were reduced to different degrees. Through the treatment of CMCS-SA-AmCS-SA-**3J** or CMCS-SA-AmCS-SA, the infiltration of inflammatory cells in the ulcer was significantly reduced. What is more, the therapeutic effect was enhanced with the increase of the concentration of **3J** in the CMCS-SA-AmCS-SA.

Combined with the results of changes in the area of oral ulcers in Figure 10A–C, if only treated with **3J**, the therapeutic effect is poor. This may be due to the slow absorption of **3J** when applied externally. Additionally, it is easily licked by rats, resulting in insufficient dosage of **3J**. In the whole treatment process, it can be seen that CMCS-SA-AmCS-SA showed good therapeutic ability for oral ulcer. Carboxymethyl chitosan and chitosan quaternary ammonium salt have good anti-inflammatory activity so that the hydrogel also has this ability. In summary, CMCS-SA-AmCS-SA-**3J** (20.0 mg/kg) has great potential for oral ulcer treatment.

## 3. Conclusions

In this paper, carboxyl chitosan, chitosan quaternary ammonium salt, and SA were used as the skeleton, and CaCl_2_ was used as a cross-linking agent to prepare an intelligent composite hydrogel. Based on the FTIR results, it is evident that CMCS-SA-AmCS-SA has abundant carboxyl, hydroxyl, and quaternary ammonium cation groups. The swelling performance of this substance varies significantly in different pH environments, indicating its ability to respond to pH changes. These findings collectively confirm the pH response ability of CMCS-SA-AmCS-SA. The hydrogel CMCS-SA-AmCS-SA has a dense honeycomb internal structure similar to sponge. That is conducive to drug loading. Additionally, it is also conducive to wound respiration and promoting wound healing. This feature also improves the water retention rate of the hydrogel, which can keep the wound in a humid environment. Additionally, the use of CMCS-SA-AmCS-SA hydrogel loaded with **3J** demonstrated a more effective anti-inflammatory response compared to either a single hydrogel or drug, resulting in up to a 50% decrease in TNF-α and IL-6 levels in inflammatory cells.

Inflammation is considered to be a key factor that is closely associated with many acute or chronic diseases. The levels of inflammatory factors fluctuate throughout the progression of oral ulcer, causing significant discomfort and inconvenience in daily life due to the associated pain. The antibacterial and anti-inflammatory experiments showed that the composite hydrogel had anti-inflammatory activity and antibacterial effect. Moreover, the results showed better anti-inflammatory effect after **3J** loading than a single hydrogel or drug treatment alone. The rat model of oral ulcer was established using phenol induction to estimate the curative effect of **3J** and complex hydrogel. The results showed that both of them had the ability to treat oral ulcers, and the compound hydrogel CMCS-SA-AmCS-SA-**3J** loaded with drugs had a better effect. Therefore, the data suggest that CMCS-SA-AmCS-SA-**3J** has the latent capacity to be developed as an external drug for the remedy of oral ulcers.

## 4. Materials and Methods

### 4.1. Materials

Carboxymethyl chitosan (BR, water soluble), chitosan quaternary ammonium salt (98.0% substitution), sodium alginate (AR, 90.0%, M/G = 1:2), anhydrous calcium chloride (for insect cell culture, for plant cell culture, ≥96.0%), and PBS Buffer (0.5M, pH 7.0) were all purchased from MACKLIN reagent. All solvents were used as received and no further purification was required.

### 4.2. Preparation of Compound 3J

**Figure 11:**
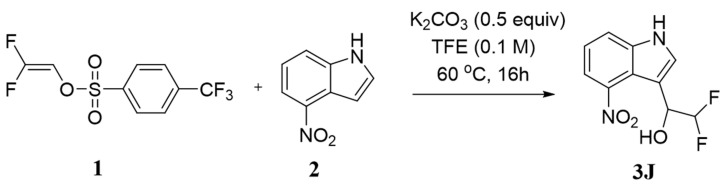
The synthetic route of **3J**.

Small molecule drugs were synthesized according to the synthesis route by Cai et al. [32]. The synthesis roadmap is as follows (Figure 11):

### 4.3. Preparation of Composite Hydrogels

#### 4.3.1. Preparation of AmCS-SA-3J

Sodium alginate (SA) and chitosan quaternium salt (AmCS) powder were accurately mixed and dissolved in warm (37 ℃) deionized water and were continuously stirred to make a homogeneous gel consisting of preliminary crosslinking of AmCS (10.0%, *w*/*v*) and SA (5.0%, *w*/*v*), and then an appropriate amount of **3J** was weighed. After being dissolved with dimethyl sulfoxide (DMSO), it was mixed into the preliminary hydrogels. CaCl_2_ (2.0%, *w*/*v*) was slowly dripped into the mixture gel with a 0.5 mm diameter syringe. At the same time, the conglomeration was continuously mixed at room temperature for 30 min to make it undergo secondary cross-linking and hardening to form AmCS-SA-**3J** hydrogel, which was filtered, washed, and freeze-dried for use as well as the blank hydrogel.

#### 4.3.2. Synthesis of Complex Hydrogels

Carboxymethyl chitosan (CMCS) and SA were accurately weighed and dissolved in deionized water at 37 °C so that the concentrations of the two were CMCS (10.0%, *w*/*v*) and SA (5.0%, *w*/*v*), respectively. Following that, the freeze-dried AmCS-SA-**3J** gel powder and an appropriate amount of **3J** solution were added and stirred until a uniform composite hydrogel was formed. CaCl_2_ (2.0%, *w*/*v*) solution was slowly added into the composite hydrogel through a syringe and stirring was continued for 1 h until CMCS-SA-AmCS-SA-**3J** composite hydrogel was formed. After forming a hydrogel, an appropriate amount of distilled water was added to clean it for 3–4 times to remove excess CaCl_2_. Similarly, a blank composite hydrogel, CMCS-SA-AmCS-SA, was prepared without the addition of drug molecules.

### 4.4. Characterization of Composite Hydrogels

#### 4.4.1. FT-IR

FT-IR was used to analyze the composite hydrogels to confirm the interactions between the components in the composite hydrogels. The composite hydrogel samples, CMCS, AmCS, and SA raw materials, were dried in a vacuum freeze dryer for more than 4 h. Using the tablet method, an appropriate amount of each sample was mixed with KBr, fully ground and pressed into thin slices, and placed on the Fourier infrared spectrometer to test the infrared spectra of each sample group, ranging from 500.0 to 4000.0 cm^−1^.

#### 4.4.2. Surface Topography Analysis

The freeze-dried hydrogel was frozen in liquid nitrogen for 10 min, then it was quenched, its section was exposed, and its surface and section were adhered upward to the copper platform, respectively. Gold was sprayed with an ion sputtering instrument for 5 min, and then the surface structure and section structure of the sample were observed by scanning electron microscopy (SEM).

#### 4.4.3. Determination of Swelling, Water Retention, and Degradation

An appropriate amount of freshly prepared composite hydrogel sample was dried in a vacuum oven at 50 °C to constant weight and was weighed and recorded as M_0_ at this time. To begin, immerse the dried samples in separate solutions of 10.0 mL Sodium Phosphate Buffer (PBS buffer) with varying pH levels (0.2 M, pH4.5, 7.4, 8.5) and ultrapure water (UPW). Following that, keep them at a constant temperature of 37 °C. At different time points, the sample was taken out at the set time point, the surface moisture was removed, and the mass at this time was measured until the composite hydrogel sample reached the saturated state, and the mass at this time was recorded as M_1_.

Water uptake (W_u_) was calculated as follows:

$$W_{u}=\frac{M_{1-}M_{0}}{M_{0}}\times 100\%$$

The composite hydrogel of a certain mass (W_i_) was weighed and evenly applied on the surface of a glass dish with a thickness of 2.0 mm at 37 °C. The mass was weighed at a predetermined time interval (n) and recorded as W_n_. We calculated the water retention rate (W_r_) corresponding to each time interval as follows.

$$W_{r}=1-\frac{W_{i}-W_{n}}{W_{i}}\times 100\%$$

The freeze-dried composite hydrogel was soaked in PBS with pH 7.4 and placed in a constant temperature incubator at 37 °C. After the swelling balance was reached, fresh PBS was replaced. At regular intervals we removed the remaining hydrogel samples, weighed and recorded their quality after freeze-drying, and replaced this with fresh PBS. The in vitro degradation rate (D_v_) of composite hydrogels was calculated as follows:

$$D_{v}=\frac{m_{c}}{m_{0}}\times 100\%$$

where m_c_ is the mass of the composite hydrogel in the degradation process and m_0_ is the initial mass of the composite hydrogel.

The above experiments were gauged three times, respectively, and the mean value obtained was the final result of the experiment [44].

#### 4.4.4. Test of Mechanical Properties of Hydrogels

The Young’s modulus of the composite hydrogel was measured using an atomic force microscope (Model: NT-MDT NTEGRA, NT-MTD, Amsterdam, The Netherlands) in contact mode using the NT-MDT and TIPS HA_NC probe. Before the test, it was necessary to prepare a hydrogel sample with a smooth, dry, and clean surface to avoid errors during the test. The sample was placed on the platform of the atomic force microscope, fixed with a clamp to ensure that the surface of the sample was parallel to the platform, the original parameters were adjusted, the sample was scanned, the coordinates of the sampling points and the data of each sampling point were recorded, the Young’s modulus of each sampling point was computed, and the viscoelasticity of the hydrogel was studied.

#### 4.4.5. Evaluation of Entrapment Efficiency (EE) and Drug Loading (DL)

The prepared composite hydrogel was dialyzed in a special dialysis bag (Flat Width: 38.0 mm; Cat. No. YA1035; Purchased from Solarbio^®^, Beijing, China) with a retention of 1000 Da and then freeze-dried in a freeze-drying machine for 24 h. An appropriate amount of freeze-dried composite hydrogel was dissolved into suspension with DMSO. We determined the wavelength corresponding to the maximum absorption peak of **3J** through full band ultraviolet spectroscopy analysis and drew a standard curve. To determine drug content, we measured the absorbance of each sample using a UV spectrophotometer at its maximum absorption wavelength. We then converted the data based on the plotted standard curve.

The expression of EE is as below:

$$\mathrm{EE}=\frac{W_{\mathrm{entrapped}}}{W_{\mathrm{total}}}\times 100\%$$

The computing formula of DL is as follows:

$$\mathrm{DL}=\frac{W_{\mathrm{entrapped}}}{W_{\mathrm{solid}}}\times 100\%$$

In the above two formulas, “W_entrapped_” is the amount of drug embedded in the composite hydrogel; “W_total_” is the total amount of drug added to the formula; “W_solid_” is the total mass of the solid components in the formula.

### 4.5. In Vitro Release Test

Using PBS (PBS Buffer:DMSO = 1.5:1) with pH 7.4 as the release medium, the freeze-dried drug-loaded complex hydrogel (equivalent to 10.0 mg of small molecule drugs) was suspended in the dialysis bag membrane (MWCO: 3500 Da; Flat Width: 44.0 mm; Lot. No. 408D0221; Purchased from Solarbio^®^), and then the bag was stored in a beaker with PBS and stirred with a magnetic stirrer at 200 rpm/min (37.0 ± 0.5 °C). At the predetermined time point, remove 2.0 mL of dialyzed solution each time, and then replenish with fresh medium to maintain the environment in the beaker. Analyze the drug release at each time point using UV spectrophotometry at a maximum absorption wavelength of **3J**.

### 4.6. In Vitro Anti-Bacterial Assay

Mid-log phase bacteria (*Escherichia coli* ATCC 25922 and *Staphylococcus aureus* ATCC 25923) were harvested and washed three times with PBS using centrifugation. A total of 1.0 mL of the bacterial cells at a consistency of 1 × 10^6^ CFU/mL were cultured in a 24-well plate containing different hydrogels for 12 h. Subsequently, the culture was placed on a Luria-Bertani (LB) solid medium and incubated at 37 °C for 12 h. The detection of bacterial growth was performed by determining the OD_600_ after incubation for 24 h [45].

### 4.7. In Vitro Anti-Inflammatory Assay

RAW 264.7 macrophages cells were cultured in DMEM containing 10% fetal bovine serum and 1.0% penicillin (100 U/mL)/streptomycin (100.0 µg/mL) in an incubator containing 5.0% CO_2_ at 37 °C. Cells were pre-treated with **3J** or different hydrogels for 2 h then exposed to LPS (1 μg/mL) for 24 h. The concentration of IL-6 and TNF-α in supernatant was measured by ELISA [46].

### 4.8. Evaluation of Oral Ulcer Animal Model

The methods mentioned in the literature were used to select SD male rats (12 weeks old, 180.0–220.0 g in weight) after adaptive feeding, and the rats were anesthetized by intraperitoneal injection with 3.0% pentobarbital sodium at a dose of 1.0 mL/100.0 g. The plastic dropper was cut to form a 4 mm diameter circle, and a cotton ball soaked with 95% phenol was inserted into it. The rat mouth was opened with tweezers and placed on the cheek for 120 s to establish a rat model of phenol-induced oral ulcer [47]. The rats were divided into Control group, Model group, CMCS-SA-AmCS-SA group, **3J** (10.0 mg/kg) group, **3J** (20.0 mg/kg) group, CMCS-SA-AmCS-SA-**3J** (5.0 mg/kg) group, CMCS-SA-AmCS-SA-**3J** (10.0 mg/kg) group, and CMCS-SA-AmCS-SA-**3J** (20.0 mg/kg) group randomly. The frequency of administration in the administration group was once a day for five days. The pH value at the ulcer was recorded before each administration, and the pH value at the wound site was also recorded in the other groups once a day. Finally, the effect of treatment was evaluated by observing the changes of oral ulcer area and the histopathology of the oral ulcer in each group.

### 4.9. H&E Staining

The samples were treated for histopathology analysis as described previously [48]. The skin tissue at ulcer was excised and fixed with 4.0% formalin. After dehydration, embedding, and dewaxing, the slices were stained with hematoxylin-eosin. After dehydration and transparency, the slices were sealed with neutral gum for observation.

### 4.10. Experimental Data Statistics and Analysis

Data were indicated as mean (±SD), and *p* < 0.05 was considered as statistical differences. The statistical analysis was carried out using the *t* test and one-way analysis of variance (ANOVA) by SPSS. The experiments were independently replicated at least three times.

## Data Availability

Data can be accessed upon request to the corresponding author.
